# Cone Density Changes After Repeated Low-Level Red Light Treatment in Children With Myopia

**DOI:** 10.1001/jamaophthalmol.2025.0835

**Published:** 2025-04-24

**Authors:** Xinyi Liao, Jifeng Yu, Yuzhuo Fan, Yixuan Zhang, Yan Li, Xuewei Li, Hongxin Song, Kai Wang

**Affiliations:** 1 Department of Ophthalmology, Peking University People’s Hospital, Beijing, China; 2 Beijing Key Laboratory of Ocular Disease and Optometry Science, Peking University People’s Hospital, Beijing, China; 3 College of Optometry, Peking University Health Science Center, Beijing, China; 4 Department of Ophthalmology, Beijing Children’s Hospital, Capital Medical University, National Center for Children’s Health, Beijing, China; 5 Institute of Medical Technology, Peking University Health Science Center, Beijing, China; 6 Beijing Tongren Eye Center, Beijing Institute of Ophthalmology, Beijing Tongren Hospital, Capital Medical University, Beijing, China; 7 Beijing Key Laboratory of Ophthalmology and Visual Sciences, Beijing, China; 8 National Engineering Research Center for Ophthalmology, Beijing, China

## Abstract

**Importance:**

Repeated low-level red light (RLRL) therapy has emerged as a potential intervention for controlling myopia progression in children. However, its long-term effects on retinal photoreceptors remain relatively unknown.

**Objective:**

To evaluate changes associated with RLRL therapy on cone photoreceptor density in children with myopia using high-resolution adaptive optics scanning laser ophthalmoscopy (AOSLO).

**Design, Setting, and Participants:**

This retrospective multicenter cohort study analyzed data collected from January to March 2024, focusing on Chinese children with myopia. All participants were recruited through questionnaires. Cone density measurements were obtained from AOSLO retinal images. Children with myopia aged 5 to 14 years recruited from the pediatric ophthalmology clinic during routine eye examinations were included in the study and assigned to the RLRL group or the control group. Inclusion criteria were spherical equivalent refraction below −6.00 diopters (D) and best-corrected visual acuity ≥20/20.

**Exposures:**

Cone density measurement with AOSLO retinal images.

**Main Outcomes and Measures:**

Cone photoreceptor density was measured along 4 retinal meridians from central fovea to 4° eccentricity on AOSLO. Fundus abnormalities were assessed using AOSLO, optical coherence tomography (OCT), and fundus photography. Image evaluators were masked to group allocation.

**Results:**

A total of 99 children with myopia were included in this analysis: 52 (97 eyes; mean [SD] age, 10.3 [1.9] years; 27 female [51.9%]) in the RLRL group and 47 (74 eyes; mean [SD] age, 9.8 [2.1] years; 25 male [53.2%]) in the control group. RLRL users showed decreased cone density within 0.5-mm eccentricity from the foveal center, most notably in the temporal region. At 0.3-mm temporal eccentricity, the RLRL group showed a mean difference of −2.1 × 10^3^ cells/mm^2^ compared with controls (95% CI, −3.68 to −0.59 × 10^3^ cells/mm^2^; *P* = .003). A total of 11 eyes exhibited abnormal low-frequency, high-brightness signals near the fovea. The odds ratio of abnormal signals in RLRL users compared with nonusers was 7.23 (95% CI, 1.15-303.45; Fisher exact test, *P* = .02). One participant had relatively small cystoid abnormalities on OCT in the ganglion cell layer, which resolved 3 months after discontinuing RLRL therapy.

**Conclusions and Relevance:**

Results of this cohort study suggest that RLRL therapy for at least 1 year was associated with reduced cone density in the paracentral fovea and other subtle retinal abnormalities in some children receiving this therapy for myopia control. These findings support the need for further research to evaluate longer-term safety of RLRL therapy in similar individuals.

## Key Points

**Question:**

Are there changes in cone density detectable in children after repeated low-level red light (RLRL) therapy given to try to control myopia?

**Findings:**

In this cohort study, among 52 children using RLRL therapy for over 1 year compared with 47 nonusers, RLRL users showed lower cone density, particularly within 0.5 mm from the retinal fovea, with abnormal drusenlike lesions detected in some of these cases.

**Meaning:**

These findings suggest that RLRL therapy may be associated with cellular-level retinal changes in macular cone cells, highlighting the importance of further studies assessing its safety for young patients with myopia, especially with long-term exposure.

## Teaser

This cohort study investigates detectable changes in cone density after repeated low-level red light therapy to control myopia in children.

## Introduction

Repeated low-level red light (RLRL) irradiation is a treatment modality that uses low-intensity red light for noncontact repeated irradiation of both eyes. RLRL therapy is being researched for inhibiting myopia progression in children and adolescents.^1,2,3,4,5,6,7^ The wavelength used in RLRL treatment is 650 ± 10 nm, with an optical power of at least 0.29 mW entering the 4-mm pupil, complying with the international safety standard for laser use.^8^ However, continuous irradiation may induce a photothermal effect and, therefore, cause retinal damage.^9^ Reports reveal that commercially available light therapy devices may theoretically cause retinal damage even when adhering to international standards.^9^ A report of retinal damage associated with RLRL treatment raised concerns about the potential harm to users.^10^

Current RLRL efficacy studies assess retinal safety via fundus photography, ultrasound, and optical coherence tomography (OCT).^11^ Jiang et al^3^ defined adverse events as short-term visual acuity loss of more than 2 lines, dark spots in the field of view, decreased best-corrected visual acuity, or structural damage in OCT scans. However, laser-associated retinal damage primarily affects the retinal pigment epithelial layer and photoreceptor cell layer,^12,13^ which may not be evident in the early stages and may only manifest as reduced photoreceptor cell density and function at the cellular level. Traditional fundus diagnostic equipment lacks resolution to observe photoreceptor cells and can only examine and diagnose the fundus at the tissue scale.

Adaptive optics scanning laser ophthalmoscopy (AOSLO) enables high-resolution retinal imaging to measure cone cell density in vivo. This study used AOSLO to compare cone photoreceptors changes in RLRL users with children with myopia who never underwent RLRL therapy.^14,15^ We acquired AOSLO images of 4 retinal meridians (superior, inferior, nasal, and temporal) from central fovea vicinity to approximately 4° retinal eccentricity to compare the differences in cone density and structure between cohorts.

## Methods

### Participants

The study protocol adhered to the tenets of the Declaration of Helsinki and was approved by the ethics review board of Peking University People’s Hospital. All participants or their legal guardians provided written informed consent. This study followed the Strengthening the Reporting of Observational Studies in Epidemiology (STROBE) reporting guidelines.

This study included participants aged 5 to 16 years with a spherical equivalent refraction no less than −6.00 diopters (D) and best-corrected visual acuity of 20/20 or better. Participants in both the control and RLRL groups were recruited by questionnaires from January 15, 2024, to March 18, 2024, with sex reported by the parents or legal guardians. The RLRL group comprised participants who underwent regular daily irradiation for more than 12 months with registered devices operating at 0.2 to 2 mW laser power. All participants treated with RLRL received a complete ophthalmic examination, and only those meeting this criterion were included in the RLRL group and underwent cone densitometry. The non-RLRL group included participants with no history of RLRL therapy exposure. Participants did not receive any financial compensation or other incentives for their participation in this study. All RLRL participants were from different medical institutions who had undergone RLRL treatment but not from a specific RLRL cohort study registered before. All participants in this study were Chinese, and all self-identified as Han Chinese.

The exclusion criteria included strabismus, amblyopia, cataracts, retinopathy, optic neuropathy, and previous ophthalmic surgery. Additionally, individuals whose images did not meet quality standards were excluded from density statistics.

During the imaging process, we collected and analyzed images from both eyes of each participant. In cases where the image quality of 1 eye was unsatisfactory, we retained the image of the contralateral eye for analysis. Consequently, the final number of eye images did not correspond directly to the number of participants, as some individuals contributed images from both eyes, whereas others provided images from only 1 eye.

All individuals underwent comprehensive ophthalmologic examinations, including best-corrected visual acuity assessment and fundus photography (Topcon Corporation). Ocular axial length was measured using a biometer (IOL Master 500 [Carl Zeiss Meditec]) to convert density measurements from angular to metric coordinates. Macular OCT scans were performed along 12 clockwise directions for each participant (Topcon Corporation).

### Apparatus

Cone photoreceptor images were acquired using the AOSLO system (Robotrak). This AOSLO system has a navigation system to help localize the imaging area. Image acquisition was performed in a dark room to ensure pupil dilation. The AOSLO system uses a multiplane independent refractor lens to help correct wavefront aberrations and ensure the acquisition of high-quality images.

### Procedures

#### Imaging

We acquired multiple small-field image sequences (approximately 2.4° × 2.4° per image) arranged in a strip pattern to cover a broader retinal area. We acquired images of the 0°, 1.5°, and 2° regions from the central fovea to the peripheral region so that the images from the central fovea to the 4° region of the periphery formed a complete strip. Each strip started from the fovea and extended toward the peripheral retina along the 4 major meridians (nasal, temporal, superior, and inferior). The acquisition process took approximately 15 to 20 minutes for each participant.

#### Montage Formation

Subregional images were integrated offline using image-processing software (Photoshop 2024 [Adobe]) to form a montage. All montage images were verified for scale and positional accuracy by comparing them with fundus photography (eFigure 1 in Supplement 1).

#### Fovea Determination and Montage Segmentation

During AOSLO, the participants were instructed to fixate on the center of the scanning raster and subsequently on each corner. The foveal center reference point for retinal coordinates was determined by identifying the center of the raster image during central fixation and comparing it with the overlapping area of the 4 images obtained during corner fixations.

The actual visual field was corrected by adjusting for each participant’s axial length using the Indiana model eye. This conversion from angular to metric coordinates (millimeters) ensured consistent comparisons across individuals, as variations in axial length affect the physical size of the retina and the visual field.

Standard montage windows of 50 × 50 μm were generated and positioned centrally on the fovea, extending along the 4 meridians (nasal, temporal, superior, and inferior) at 100-μm intervals. In instances where the montage window encountered a junction line between 2 original images, image quality defects, or occlusion by large blood vessels, the sampling area was shifted to an appropriate position (centripetal or centrifugal by 25 μm). These shifted montage window positions were subsequently relabeled to maintain accurate spatial referencing.

#### Cone Photoreceptor Quantification

An automated algorithm was employed to label and enumerate cone photoreceptors within each montage window. The results were then manually reviewed and edited by the researcher to ensure accurate final cone-cell positioning. Given that cone photoreceptor density is directly correlated with axial length, we implemented an algorithmic correction for the position and true size of each segmentation pair on the basis of individual axial length measurements.

Following the correction for position and pane size, we retained the final pane positions without further averaging or interpolation. The location of all cone photoreceptors within each 50 × 50-μm pane was automatically identified using a custom cell image segmentation algorithm (based on a semiautomatic ImageJ [National Institutes of Health, Wayne Rasband] training model). Researchers then manually edited the cone photoreceptor identification within each pane to rectify any discrepancies in position or number. During this process, the brightness and contrast of the cone photoreceptor images were enhanced to facilitate accurate manual correction.

### Statistical Analysis

To test the difference in cell densities near the central fovea between the RLRL group and the non-RLRL group, after verifying the normal distribution of the overall data, we counted the cone densities of the participants near 0.20, 0.30, 0.40, 0.50, 0.60, 0.80, 1.00, and 1.20 mm and used independent-samples *t* test analyses (SPSS, version 26.0 [IBM]). One-sided *P* values were used for RLRL vs non-RLRL group comparisons given our directional hypothesis that RLRL exposure may be associated with reduced cone density, whereas 2-sided *P* values were used for other analyses. A *P* value <.05 was considered statistically significant.

To better observe the cell density near the fovea, we performed measurements at 0.10-mm intervals within 0.60 mm from the fovea and at 0.20-mm intervals between 0.60 mm and 1.20 mm from the fovea. Owing to extremely high cone density at the macular fovea, accurate counting of cone density at 0.10 mm from or at the central fovea was not possible. The apparent lower density in the images at this distance resulted from inadequate resolution. Consequently, data on the cell density at the first sampling point near the central fovea (approximately 0.1 mm) were excluded to avoid excessive errors caused by low-resolution counting (eFigure 2 in Supplement 1).

To account for varying power and daily usage times in RLRL users, the RLRL group was divided evenly into 3 subgroups on the basis of total energy: <270 J, 270 to 430 J, and ≥430 J. The total exposure energy was calculated as RLRL use power (watt) × daily use time (second) × days of use. We used 1-way analysis of variance to analyze the association of different total red light exposures with the participants’ cell density. In addition, for the case-statistical analysis of participants with subjective symptoms, we analyzed the cell density in each orientation in the range of 0.2 to 0.5 mm compared with the overall cell density of the individual. This analysis was performed using single-sample *t* tests.

Three independent researchers (Y.F., Y.Z., and X. Li) assessed abnormal cell clusters with high-brightness and low-frequency signals near the fovea in randomized, deidentified AOSLO fundus images. The results were compiled and analyzed after group identity was revealed.

## Results

### Basic Data of the RLRL Group and Non-RLRL Group

A total of 69 RLRL users underwent AOSLO examinations. Seven participants reported subjective symptoms during RLRL therapy (eTable 1 in the Supplement). Three participants had used RLRL for more than 12 months but were not included in the density count due to the poor quality of the AOSLO images. An additional 14 participants were not included in the overall statistics for not using RLRL for more than 12 months. After exclusions, 52 participants (97 eyes; mean [SD] age, 10.3 [1.9] years; 27 female [51.9%]; 25 male [48.1%]) met the RLRL group criteria. The control group included 47 participants (74 eyes; mean [SD] age, 9.8 [2.1] years; 22 female [46.8%]; 25 male [53.2%]) who never underwent RLRL therapy. Details of RLRL instruments are provided in eTable 2 in Supplement 1.

No differences between groups in baseline characteristics were identified, including spherical equivalent refraction (mean [SD], RLRL: −1.51 [1.44] D; control: −1.76 [1.05] D), mean macular thickness (mean [SD], RLRL: 223 [15.52] μm; control: 220 [15.92] μm), and ocular axis length (mean [SD], RLRL: 24.33 [0.94] mm; control: 24.34 [0.99] mm). Complete baseline data are provided in eTable 3 in Supplement 1.

### Differences in Paracentral Foveal Cone Density With RLRL Use

All participants showed a continuous decrease in cone density from the central fovea (0.2 mm) toward the periphery in all 4 directions, consistent with normal human retinal topology.^16^

**Figure 1.**
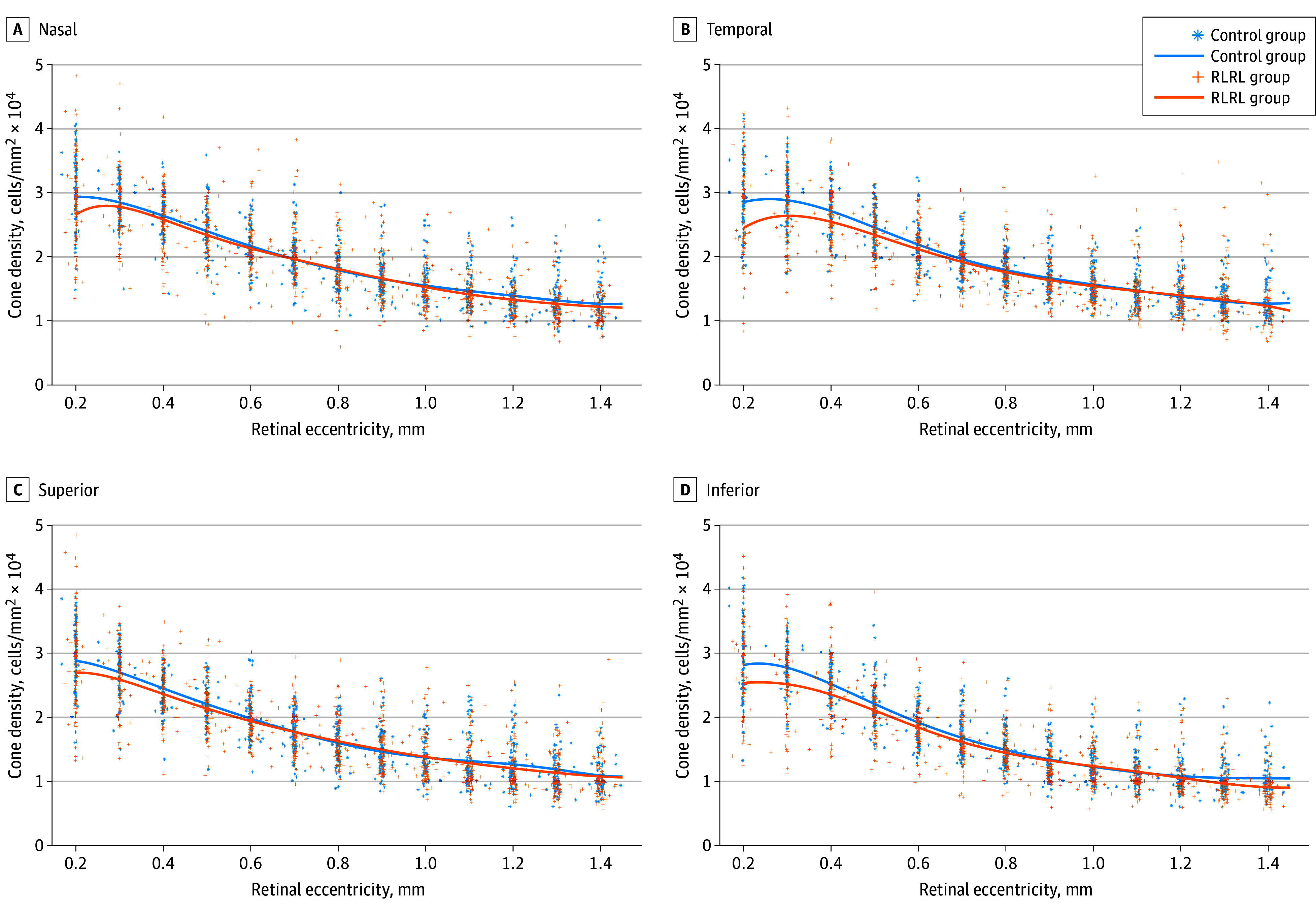
Distance-Density Distribution Plots of Paracentral Foveal Cone Photoreceptors in Participants Along Nasal, Temporal, Superior, and Inferior Meridians RLRL indicates repeated low-level red light.

RLRL users showed lower cone density than nonusers, particularly within 0.5 mm from the retinal fovea. At 0.3-mm temporal eccentricity, the RLRL group showed a mean difference of −2.1 × 10^3^ cells/mm^2^ compared with controls (95% CI −3.68 to −0.59 × 10^3^ cells/mm^2^; *P* = .003). Detailed cone densities at different eccentricities are presented in Figure 1 and Table 1.

**Table 1.** Cone Density Distributions of the Repeated Low-Level Red Light (RLRL) Group and Control Group

Region	Distance from fovea, mm^a^							
	0.2	0.3	0.4	0.5	0.6	0.8	1.0	1.2
**Nasal**								
RLRL, mean (SD)	28.0 (0.65)	27.8 (0.49)	25.6 (0.44)	23.2 (0.43)	21.7 (0.45)	17.8 (0.39)	15.0 (0.30)	13.2 (0.25)
Non-RLRL, mean (SD)	30.1 (0.56)	28.7 (0.36)	26.4 (0.33)	23.8 (0.38)	21.6 (0.35)	17.9 (0.35)	15.6 (0.33)	13.7 (0.32)
Mean difference (95% CI)	−2.13 (−4.04 to −0.22)	−0.98 (−2.35 to 0.38)	−0.81 (−2.04 to 0.42)	−0.68 (−1.98 to 0.63)	0.10 (−1.18 to 1.39)	−0.04 (−1.21 to 1.13)	−0.62 (−1.60 to 0.36)	−0.48 (−1.38 to 0.43)
*P* value	.02	.07	.09	.15	.44	.47	.11	.15
**Temporal**								
RLRL, mean (SD)	27.5 (0.69)	27.1 (0.52)	25.4 (0.44)	23.2 (0.37)	21.3 (0.34)	17.9 (0.34)	15.4 (0.35)	14.1 (0.42)
Non-RLRL, mean (SD)	29.4 (0.52)	29.3 (0.46)	27.0 (0.39)	24.5 (0.39)	21.9 (0.37)	18.0 (0.26)	15.5 (0.24)	13.8 (0.26)
Mean difference (95% CI)	−1.77 (−3.76 to 0.23)	−2.14 (−3.68 to −0.59)	−1.67 (−2.98 to −0.35)	−1.29 (−2.44 to −0.14)	−0.55 (−1.67 to 0.56)	−0.07 (−1.02 to 0.89)	−0.15 (−1.11 to 0.82)	0.20 (−0.88 to 1.28)
*P* value	.04	.003	.007	.01	.17	.45	.38	.36
**Superior**								
RLRL, mean (SD)	28.0 (0.66)	26.0 (0.48)	23.8 (0.40)	21.6 (0.38)	19.2 (0.31)	16.1 (0.35)	13.8 (0.39)	12.1 (0.38)
Non-RLRL, mean (SD)	29.4 (0.52)	27.3 (0.36)	24.2 (0.29)	22.0 (0.30)	20.1 (0.37)	16.0 (0.33)	13.6 (0.31)	12.6 (0.37)
Mean difference (95% CI)	−1.35 (−3.24 to 0.55)	−1.32 (−2.62 to −0.03)	−0.44 (−1.52 to 0.63)	−0.32 (−1.45 to 0.81)	−0.85 (−1.93 to 0.23)	−0.11 (−0.94 to 1.16)	0.20 (−0.91 to 1.33)	−0.51 (−1.70 to 0.69)
*P* value	.08	.02	.21	.29	.06	.42	.36	.20
**Inferior**								
RLRL, mean (SD)	27.9 (0.69)	26.0 (0.55)	23.8 (0.47)	21.0 (0.38)	18.0 (0.35)	14.6 (0.31)	12.4 (0.31)	10.7 (0.31)
Non-RLRL, mean (SD)	29.3 (0.60)	28.0 (0.33)	25.1 (0.38)	22.4 (0.41)	18.7 (0.30)	15.1 (0.33)	12.2 (0.27)	10.8 (0.28)
Mean difference (95% CI)	−1.35 (−3.39 to 0.69)	−1.89 (−3.24 to −0.45)	−1.31 (−2.61 to −0.02)	−1.37 (−2.65 to 0.11)	−0.70 (−1.72 to 0.32)	−0.52 (−1.50 to 0.45)	0.19 (−0.74 to 1.11)	−0.09 (−1.04 to 0.86)
*P* value	.09	.004	.02	.02	.09	.15	.34	.43

^a^ The unit of density is 10^3^/mm^2^.

### Total RL Exposure and Paracentral Foveal Cone Density in RLRL Users

When comparing cone density at 0.20 to 0.50 mm across different meridians among participants with varying RLRL exposures (<270 J, 270-430 J, and ≥430 J), the differences between groups were small, with overlapping 95% CIs (detailed data provided in eTable 4 in Supplement 1).

### Subjective Symptoms and Paracentral Foveal Cone Density in RLRL Users

Within RLRL users, a total of 7 participants reported subjective symptoms, with 4 participants (4 of 52 [7.7%]) using RLRL regularly for more than 12 months and 3 participants (3 of 14 [21.4%]) using RLRL for less than 12 months (eTables 1 and 5 in Supplement 1). Compared with asymptomatic participants in the RLRL group, analysis of case-specific parafoveal cone densities within 0.20 to 0.50 mm across different orientations revealed significantly lower cell densities in most orientations for 3 of these symptomatic participants. However, 2 symptomatic participants (patient 3 and 6) (eTables 1 and 5 in Supplement 1) did not exhibit lower cell densities than the mean cell density in the RLRL group.

### Cases of Abnormal Cell Clusters in the Proximal Fovea

**Figure 2.**
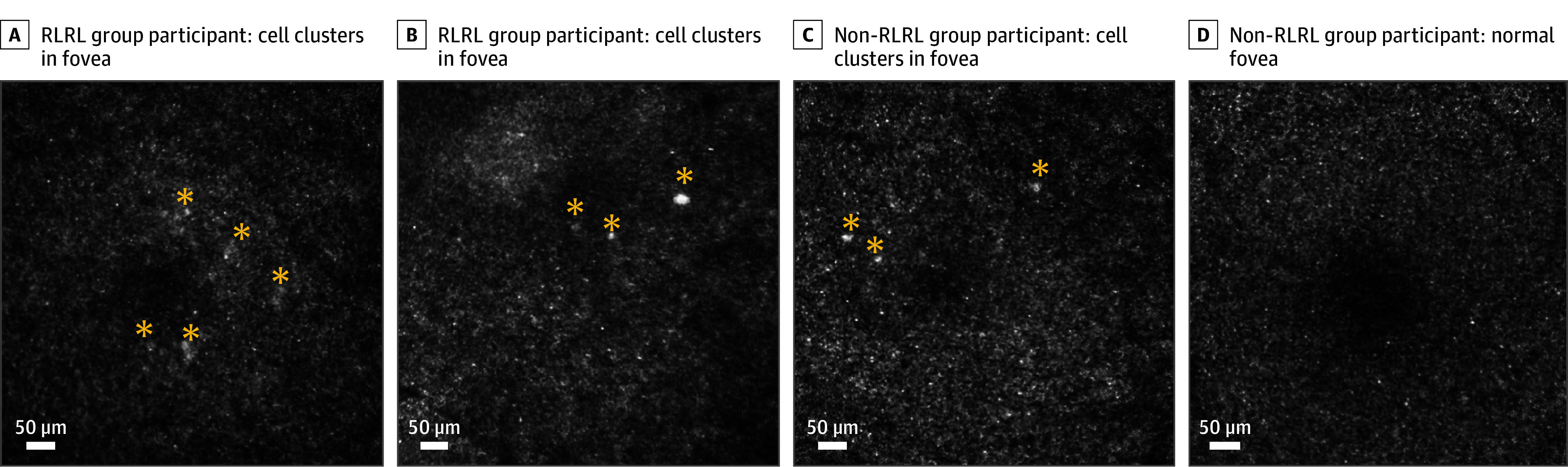
Adaptive Optics Scanning Laser Ophthalmoscopy (AOSLO) Images AOSLO images showing abnormal low-frequency high-intensity cell clusters in the fovea (A-C, yellow asterisks) compared with a normal fovea (D). Complete set of images is detailed in eFigure 3 in Supplement 1. RLRL indicates repeated low-level red light.

Eleven eyes of 10 participants exhibited low-frequency, high-brightness abnormal signals within 2.4° of the fovea (Figure 2, eFigure 3 in Supplement 1, and Table 2): 7 from long-term RLRL users (>12 months; 7 of 97 eyes [7.2%]), 3 from short-term users (<12 months; 3 of 28 eyes [10.7%]), and 1 from a nonuser (1 of 74 eyes [1.4%]). The odds ratio of abnormal signals in RLRL users compared with nonusers was 7.23 (95% CI, 1.15-303.45; Fisher exact test, *P* = .02), demonstrating a higher occurrence of abnormal signals in RLRL users. None reported visual symptoms. The abnormal signal observed is similar to the known manifestation of drusenlike damage in AOSLO images.^17,18,19^

**Table 2.** Basic Information of Participants Presenting With Abnormal Low-Frequency, High-Intensity Cell Clusters

Patient	Sex	Eye	Age, y	RLRL power, mW	Usage duration, mo	Axial length, mm	Spherical equivalent refraction, D
**RLRL ≥12 mo**							
1	M	OD	9.6	2.00	12	25.54	−2.00
2	M	OD	11.8	0.35	37	24.80	−2.00
3	F	OS	13.2	0.90	32	25.93	−2.00
4	M	OS	7.8	0.37	24	23.48	−0.50
5	M	OD	9.8	2.00	35	23.09	−1.25
		OS				23.20	−1.25
6	M	OD	10.7	1.50	24	24.80	−1.25
**RLRL <12 mo**							
7	M	OS	11.8	1.50	11	25.50	−1.00
8^a^	F	OD	7.6	2.00	3	22.77	−0.75
9	F	OS	10.3	1.50	4	24.47	−2.00
**Non-RLRL**							
10	F	OS	5.0	NA	NA	24.21	−2.25

Abbreviations: D, diopter; F, female; M, male; NA, not applicable; RLRL, repeated low-level red light.

^a^ Fifteen minutes of use per day, exceeded the recommended duration of use.

### Cystoid Cavity Within the Ganglion Cell Layer after RLRL Treatment

**Figure 3.**
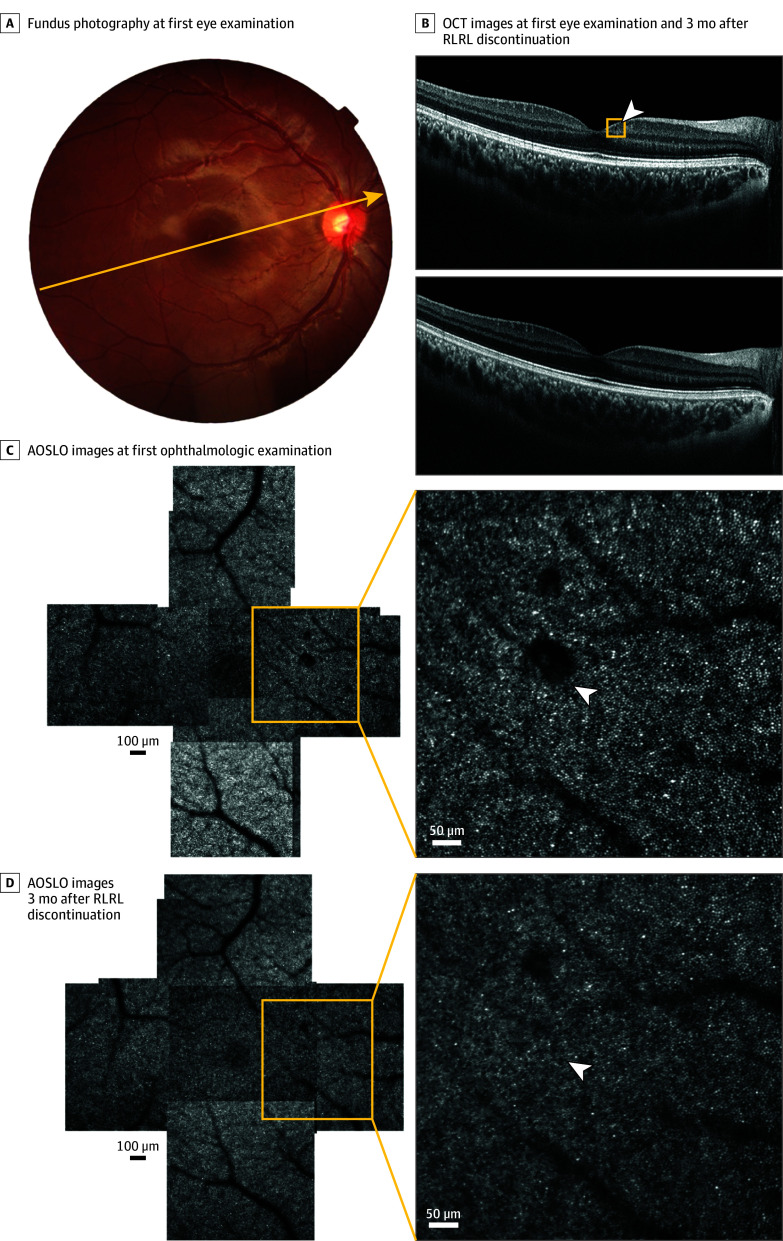
Macular Foveal Cone Images in the Optical Coherence Tomography (OCT) of Repeated Low-Level Red Light (RLRL) Results, Including Baseline and Follow-Up A, Fundus photography and a schematic cross-section of the OCT scans of the participant (yellow arrow). B, OCT images of the participant’s initial eye examination (top) and 3 months after discontinuation of RLRL (bottom). The white arrowhead in the yellow box shows the cystoid cavity in ganglion cell layer. C, Adaptive optics scanning laser ophthalmoscopy (AOSLO) images show montage formation near the fovea at the participant’s initial ophthalmologic examination. The white arrowhead shows the cystoid cavity in ganglion cell layer. D, AOSLO image of the participant’s review image after discontinuation of RLRL for 3 months. The white arrowhead shows the corresponding position of the previous cystoid cavity.

On scrutiny of each participant’s OCT images, we identified 1 individual in the RLRL group with potentially relevant alterations (Figure 3). These manifested as small cystoid cavities within the ganglion cell layer and appeared as cell clusters on the corresponding AOSLO images. This RLRL participant had regularly undergone RLRL therapy at the time of the OCT and AOSLO scans and had not experienced any subjective symptoms.

To investigate whether the changes in OCT and AOSLO images were related to RLRL therapy use and to avoid changes in images due to physiological variations or errors, we obtained consent from the participant’s guardian to completely discontinue RLRL therapy. OCT and AOSLO images were reviewed after a 3-month period.

At the 3-month follow-up, the neurofibrillary retinal cystoid cavity had disappeared from the OCT image, and the shadows in the AOSLO images had also shrunk.

## Discussion

Myopia, with spherical equivalent refraction of −0.5 D or more negative, affects more than 50% of children and adolescents in East Asia, with 10% to 20% having high myopia in excess of −6 D.^20,21,22,23,24^ The global prevalence of myopia is projected to reach 49.8% by 2050.^25^ Current control methods including outdoor activities,^26,27^ atropine eye drops,^28,29^ and orthokeratology lenses^30^ have shown varying efficacy in slowing myopia progression. Recent studies on RLRL therapy have revealed promising results in controlling ocular axial growth compared with traditional methods.^1,2,4^

Despite its possible efficacy, the mechanism of RLRL therapy remains unclear. Proposed theories include increased choroidal thickness and altered scleral collagen crosslinking.^31,32^ However, concerns about retinal safety have been raised, with both case reports of retinal damage^10^ and theoretical calculations suggesting potential risks not only in the treatment of myopia^9^ but more recently when used to treat age-related macular degeneration.^33,34^

Our analysis revealed decreased parafoveal cone photoreceptor densities in long-term RLRL users, suggesting a potential associated risk of cone photoreceptor loss with RLRL therapy. Although subjective symptoms often correlated with reduced cone photoreceptor density, the normal cone density in 2 symptomatic participants could be explained by 2 factors: their pretreatment density levels were unknown and might have been initially higher, and our AOSLO measurements could not differentiate between red and green cones, potentially missing selective changes in red cone populations that could explain the symptoms. The lack of correlation between total red light exposure dose and cone density may reflect individual variations in retinal sensitivity or limitations in exposure quantification.

Examination of cone photoreceptor images revealed a higher incidence of abnormal low-frequency, high-brightness cell signals near the fovea among RLRL users, resembling early drusenlike changes.^17,19^ Drusen are subretinal deposits, usually considered early signs of age-related retinal changes typically observed in middle-aged and older populations, and marked drusen are usually a sign of age-related macular degeneration.^35^ Although minimal drusen may occur in normal eyes,^18^ their increased prevalence in RLRL users warrants attention. The case of reversible cystoid cavities in 1 participant’s ganglion cell layer suggests that RLRL therapy may be associated with different retinal layers, potentially exceeding tissue tolerance limits.

One participant showed cystoid cavities in the ganglion cell layer during RLRL therapy. Sequential OCT imaging documented the presence of these cavities and their complete resolution 3 months after discontinuing RLRL therapy. Although baseline OCT images before RLRL therapy were not available, the temporal association between RLRL discontinuation and cavity resolution was noted. Light injury may affect different retinal layers based on laser duration, wavelength, and power. Red lights are considered safer, whereas blue lights have been associated with inner neurosensory retina.^36^ Our findings suggest that RLRL therapy may exceed tissue tolerance limits, causing unexpected ganglion cell layer damage.

### Limitations

This study has several limitations. First, this study relied on AOSLO imaging for structural changes, lacking comprehensive functional evaluations such as color vision assessment, microperimetry, and multifocal electroretinography. Second, the retrospective design lowers the ability to infer the causal relationship between RLRL therapy and observed retinal changes. Although we found an association between RLRL use and reduced cone density, further studies are still needed to determine a causal relationship and whether these retinal changes are progressive or stabilized. Even with strict exclusion criteria, potential confounding variables like genetic predispositions or environmental factors affecting retinal characteristics cannot be fully ruled out. Third, RLRL device diversity and adherence differences may introduce bias, and this study cannot comprehensively analyze the correlation between these retinal changes and myopia control efficacy. The unclear dose-response relationship complicates result interpretation.

## Conclusions

In this cohort study, we found that children receiving RLRL therapy showed lower cone density in the paracentral fovea compared with nonusers, with some participants displaying subtle retinal abnormalities. RLRL therapy administered for at least 1 year was associated with reduced cone density in the paracentral fovea and other subtle retinal abnormalities in some children receiving this therapy for myopia control compared with evaluation of nonusers of RLRL therapy. Although RLRL therapy might control the progression of myopia, further studies are needed to provide more definitive information regarding longer-term efficacy and safety. The AOSLO findings from this investigation support further evaluation of the risk-benefit balance of RLRL therapy in pediatric patients with myopia and potentially the inclusion of additional studies that include AOSLO evaluations as part of the efficacy and safety of RLRL therapy.

## Notes

Supplement 1.
eTable 1. Basic Information of RLRL Users Who Reported Subjective Symptoms
**eTable 2.** The Manufacturer’s Brand of the Device Used for RLRL Therapy and the Power Indicated **eTable 3.** Mean Baseline Characteristics **eTable 4.** ANOVA Test and Post Hoc Analysis for Cone Density **eTable 5.** One-Sample *t*-Test for Optic Cone Density **eFigure 1.** Montage Formation in Fundus Images and OCT Images **eFigure 2.** Cone Images at Different Eccentricities **eFigure 3.** AOSLO Pictures of Abnormal Cell Clusters
eReferences

Supplement 2.
Data Sharing Statement.
