# Supplementary material for: Cone Density Changes After Repeated Low-Level Red Light Treatment in Children With Myopia
Source: JAMA Ophthalmol. 2025 Apr 24;143(6):480–8. doi: 10.1001/jamaophthalmol.2025.0835 (PMC12022868; doi:10.1001/jamaophthalmol.2025.0835)
Supplement: Supplement 1. — eTable 1. Basic Information of RLRL Users Who Reported Subjective Symptoms eTable 2. The Manufacturer’s Brand of the Device Used for RLRL Therapy and the Power Indicated eTable 3. Mean Baseline Characteristics eTable 4. ANOVA Test and Post Hoc Analysis for Cone Density eTable 5. One-Sample t-Test for Optic Cone Density eFigure 1. Montage Formation in Fundus Images and OCT Images eFigure 2. Cone Images at Different Eccentricities eFigure 3. AOSLO Pictures of Abnormal Cell Clusters eReferences [file jamaophthalmol-e250835-s001.pdf]

Supplementary Online Content

Liao X, Yu J, Fan Y, et al. Cone density changes after repeated low-level red light treatment in children with myopia. *JAMA Ophthalmol*. Published online April 24, 2025.
doi:10.1001/jamaophthalmol.2025.0835

eTable 1. Basic Information of RLRL Users Who Reported Subjective Symptoms

eTable 2. The Manufacturer's Brand of the Device Used for RLRL Therapy and the Power Indicated

eTable 3. Mean Baseline Characteristics

eTable 4. ANOVA Test and Post Hoc Analysis for Cone Density

eTable 5. One-Sample *t*-Test for Optic Cone Density

eFigure 1. Montage Formation in Fundus Images and OCT Images

eFigure 2. Cone Images at Different Eccentricities

eFigure 3. AOSLO Pictures of Abnormal Cell Clusters

eReferences

This supplementary material has been provided by the authors to give readers additional information about their work.

© 2025 Liao X et al. *JAMA Ophthalmol*.

eTables

eTable 1. Basic information of RLRL users who reported subjective symptoms

	Sex	Age /years	Power /mW	Usage Duration /months	Subjective Symptom(s)
1	F	11.7	2	36	Flashes in vision
2	M	12.3	2	21	Flashes in vision
3	F	11.5	0.35	36	Prolonged afterimage
4	F	10.8	1.5	29	Green vision for short time
5	M	9.1	2	3	Vision discoloration, flashes in vision, Prolonged afterimage
6	F	9.3	1.5	7	Green vision for short time
7	M	10.1	2	4	Prolonged afterimage

© 2025 Liao X et al. *JAMA Ophthalmol*.

eTable 2. The manufacturer's brand of the device used for RLRL therapy and the power indicated. [1]

Device and manufacturer	Wavelength (nm)	Power
Eyerising, Suzhou Xuanjia Optoelectronics Technology [2,3,4,5]	650	0.29 mW (through 4 mm pupil), 1.63 mW, 2 mW
Sky-n1201, Beijing Ming Ren Shi Kang, Science and Technology Co., Ltd. [6]	650	0.37 mW, 0.6 mW, 1.2 mW
Longda, Jilin Longda Optoelectronics Technology, Jilin, China [7]	650	0.35 mW
Yishiliang, Hunan Yuanliang Optoelectronics Technology [8]	650	0.39mW, 0.9 mW, 1.5 mW

© 2025 Liao X et al. *JAMA Ophthalmol*.

eTable 3. Mean Baseline Characteristics

	RLRL Group (n=52, 97 eyes)	Control Group (n=47, 74 eyes)	Mean Differences	95% CI	*P* Value
Age (years)	10.3 ± 1.9 (5.6 to 10.3)	9.8 ± 2.1 (5.0 to 15.8)	0.52	-0.29 to 1.33	.21
Spherical Equivalent (diopters)	-1.51 ± 1.44 (-6.00 to +0.75)	-1.76 ± 1.05 (-4.50 to 0.00)	0.25	-0.10 to 0.60	.16
Macular Thickness (μm)	223 ± 15.52 (191 to 262)	220 ± 15.92 (182 to 259)	3.16	-1.62 to 7.94	.19
Ocular Axis Length (mm)	24.33 ± 0.94 (22.84 to 26.9)	24.34 ± 0.99 (21.6 to 26.6 2)	-0.11	-0.30 to 0.28	.94

© 2025 Liao X et al. *JAMA Ophthalmol*.

eTable 4. ANOVA test and post hoc analysis for cone density

A. ANOVA test p-value for cone density within 0.20–0.50 mm in individuals with different RLRL exposure doses.

Distance/ mm	0.20	0.30	0.40	0.50
**Nasal**	0.780	0.311	0.351	0.136
**Temporal**	0.322	0.622	0.174	0.310
**Superior**	0.115	0.275	0.194	0.526
**Inferior**	0.679	0.664	0.877	0.710

© 2025 Liao X et al. *JAMA Ophthalmol*.

B. Post hoc test of ANOVA test of optic cone cell density at 0.20 mm in all directions in populations with different RLRL exposure doses. Group a: exposure dose <270 J, group b: 270 ≤ exposure dose <430 J, group c: exposure dose ≥430 J.

Direction	Group	To Group	Mean Differences×10^3 cells/mm^2	95% CI×10^3 cells/mm^2	*P* Value
**Nasal**	a	b	1.08	-2.37 to 4.53	.54
		c	1.02	-2.28 to 4.32	.54
	b	a	-1.08	-4.53 to 2.37	.54
		c	-0.05	-3.32 to 3.21	.97
	c	a	-1.02	-4.32 to 2.28	.54
		b	0.05	-3.21 to 3.32	.97
**Temporal**	a	b	-0.81	-4.49 to 2.88	.66
		c	1.73	-1.81 to 5.26	.33
	b	a	0.81	-2.88 to 4.49	.66
		c	2.53	-0.89 to 5.96	.15
	c	a	-1.73	-5.26 to 1.81	.33
		b	-2.53	-5.96 to 0.89	.15
**Superior**	a	b	-0.40	-3.91 to 3.11	.82
		c	2.74	-0.58 to 6.05	.10
	b	a	0.40	-3.11 to 3.91	.82
		c	3.14	-0.14 to 6.41	.06
	c	a	-2.74	-6.05 to 0.58	.10
		b	-3.14	-6.41 to 0.14	.06
**Inferior**	a	b	-0.79	-4.46 to 2.88	.67
		c	0.74	-2.74 to 4.23	.67
	b	a	0.79	-2.88 to 4.46	.67
		c	1.53	-1.92 to 4.99	.38
	c	a	-0.74	-4.23 to 2.74	.67
		b	-1.53	-4.99 to 1.92	.38

© 2025 Liao X et al. *JAMA Ophthalmol*.

C. Post hoc test of ANOVA test of optic cone cell density at 0.30 mm in all directions in populations with different RLRL exposure doses. Group a: exposure dose <270 J, group b: 270 ≤ exposure dose <430 J, group c: exposure dose ≥430 J.

Direction	Group	To Group	Mean Differences×10^3 cells/mm^2	95% CI×10^3 cells/mm^2	*P* Value
**Nasal**	a	b	1.82	-0.92 to 4.56	.19
		c	1.82	-0.79 to 4.42	.17
	b	a	-1.82	-4.56 to 0.92	.19
		c	0.00	-2.52 to 2.52	1.00
	c	a	-1.82	-4.42 to 0.79	.17
		b	0.00	-2.52 to 2.52	1.00
**Temporal**	a	b	0.34	-2.6 to 3.29	.66
		c	1.29	-1.51 to 4.09	.33
	b	a	-0.34	-3.29 to 2.6	.66
		c	0.95	-1.76 to 3.65	.15
	c	a	-1.29	-4.09 to 1.51	.33
		b	-0.95	-3.65 to 1.76	.15
**Superior**	a	b	2.10	-0.48 to 4.68	.11
		c	0.98	-1.46 to 3.43	.43
	b	a	-2.10	-4.68 to 0.48	.11
		c	-1.11	-3.48 to 1.25	.35
	c	a	-0.98	-3.43 to 1.46	.43
		b	1.11	-1.25 to 3.48	.35
**Inferior**	a	b	0.36	-2.6 to 3.32	.81
		c	1.21	-1.59 to 4.01	.39
	b	a	-0.36	-3.32 to 2.6	.81
		c	0.85	-1.86 to 3.56	.54
	c	a	-1.21	-4.01 to 1.59	.39
		b	-0.85	-3.56 to 1.86	.54

© 2025 Liao X et al. *JAMA Ophthalmol*.

D. Post hoc test of ANOVA test of optic cone cell density at 0.40 mm in all directions in populations with different RLRL exposure doses. Group a: exposure dose <270 J, group b: 270 ≤ exposure dose <430 J, group c: exposure dose ≥430 J.

Direction	Group	To Group	Mean Differences×10^3 cells/mm^2	95% CI×10^3 cells/mm^2	*P* Value
**Nasal**	a	b	0.64	-1.69 to 2.97	.59
		c	1.59	-0.62 to 3.8	.16
	b	a	-0.64	-2.97 to 1.69	.59
		c	0.96	-1.25 to 3.17	.39
	c	a	-1.59	-3.8 to 0.62	.16
		b	-0.96	-3.17 to 1.25	.39
**Temporal**	a	b	2.23	-0.12 to 4.57	.06
		c	1.07	-1.14 to 3.28	.34
	b	a	-2.23	-4.57 to 0.12	.06
		c	-1.15	-3.34 to 1.03	.30
	c	a	-1.07	-3.28 to 1.14	.34
		b	1.15	-1.03 to 3.34	.30
**Superior**	a	b	1.85	-0.27 to 3.98	.09
		c	0.43	-1.58 to 2.43	.67
	b	a	-1.85	-3.98 to 0.27	.09
		c	-1.43	-3.43 to 0.58	.16
	c	a	-0.43	-2.43 to 1.58	.67
		b	1.43	-0.58 to 3.43	.16
**Inferior**	a	b	0.52	-1.97 to 3	.68
		c	-0.05	-2.39 to 2.3	.97
	b	a	-0.52	-3 to 1.97	.68
		c	-0.56	-2.9 to 1.78	.63
	c	a	0.05	-2.3 to 2.39	.97
		b	0.56	-1.78 to 2.9	.63

© 2025 Liao X et al. *JAMA Ophthalmol*.

E. Post hoc test of ANOVA test of optic cone cell density at 0.50 mm in all directions in populations with different RLRL exposure doses. Group a: exposure dose <270 J, group b: 270 ≤ exposure dose <430 J, group c: exposure dose ≥430 J.

Direction	Group	To Group	Mean Differences×10^3 cells/mm^2	95% CI×10^3 cells/mm^2	*P* Value
**Nasal**	a	b	2.22	-0.03 to 4.47	.05
		c	1.51	-0.57 to 3.6	.15
	b	a	-2.22	-4.47 to 0.03	.05
		c	-0.70	-2.83 to 1.43	.51
	c	a	-1.51	-3.6 to 0.57	.15
		b	0.70	-1.43 to 2.83	.51
**Temporal**	a	b	1.55	-0.45 to 3.54	.13
		c	0.71	-1.12 to 2.55	.44
	b	a	-1.55	-3.54 to 0.45	.13
		c	-0.83	-2.71 to 1.04	.38
	c	a	-0.71	-2.55 to 1.12	.44
		b	0.83	-1.04 to 2.71	.38
**Superior**	a	b	1.13	-0.89 to 3.16	.27
		c	0.39	-1.54 to 2.33	.69
	b	a	-1.13	-3.16 to 0.89	.27
		c	-0.74	-2.66 to 1.17	.44
	c	a	-0.39	-2.33 to 1.54	.69
		b	0.74	-1.17 to 2.66	.44
**Inferior**	a	b	0.58	-1.49 to 2.64	.58
		c	-0.20	-2.16 to 1.77	.84
	b	a	-0.58	-2.64 to 1.49	.58
		c	-0.77	-2.7 to 1.15	.43
	c	a	0.20	-1.77 to 2.16	.84
		b	0.77	-1.15 to 2.7	.43

© 2025 Liao X et al. *JAMA Ophthalmol*.

eTable 5. One-sample t-test for optic cone density: participants in the RLRL group who reported subjective symptoms in different directions in the range of 0.20-0.50 mm.

Symptomatic Subjects	Cone Density	Difference [95%CI]	Cone Density	Difference [95%CI]	Cone Density	Difference [95%CI]	Cone Density	Difference [95%CI]
Distance/mm	0.2		0.3		0.4		0.5	
1								
Nasal	16.2	-11.8 [-13.1 to -10.4]	18.7	-9.10 [-10.1 to -8.1]	18.7	-6.96 [-7.87 to -6.06]	16.4	-6.75 [-7.64 to -5.86]
Temporal	9.0	-18.5 [-19.9 to -17.1]	16.0	-11.1 [-12.2 to -10.0]	27.3	1.92 [1.00 to 2.84]	23.5	0.26 [-0.52 to 1.03]
Superior	14.2	-13.8 [-15.2 to -12.4]	17.3	-8.68 [-9.67 to -7.69]	17.9	-5.91 [-6.74 to -5.08]	17.9	-3.78 [-4.58 to -2.98]
Inferior	18.5	-9.47 [10.90 to -0.80]	16.2	-9.79 [-10.9 to -8.66]	18.1	-5.78 [-6.75 to -4.82]	14.6	-6.37 [-7.17 to -5.57]
2								
Nasal	28.2	0.22 [-1.12 to 1.57]	25.2	-2.55 [-3.59 to -1.52]	23.8	-1.82 [-2.73 to -0.92]	21.4	-1.82 [-2.71 to -0.93]
Temporal	24.6	-2.92 [-4.36 to -1.48]	26.4	-0.73 [-1.84 to 0.38]	24.4	-0.97 [-1.89 to -0.05]	23.8	0.57 [-0.20 to 1.35]
Superior	29.3	1.24 [-0.01 to 2.62]	22.0	-3.97 [-4.96 to -2.98]	24.0	0.23 [-0.60 to 1.06]	22.5	0.85 [0.05 to 1.65]
Inferior	26.0	-1.93 [-3.36 to -0.51]	25.4	0.63 [-1.77 to 0.51]	23.4	-0.43 [-1.40 to 0.53]	20.4	-0.64 [-1.44 to 0.17]
3								
Nasal	29.2	1.15 [-0.02 to 2.50]	27.6	-0.20 [-1.24 to 0.84]	25.0	-0.66 [-1.57 to 0.25]	21.4	-1.83 [-2.72 to -0.94]
Temporal	29.0	1.42 [-0.02 to 2.86]	30.0	2.83 [1.72 to 3.94]	24.8	-0.58 [-1.50 to 0.34]	22.0	-1.23 [-2.01 to -0.46]

© 2025 Liao X et al. *JAMA Ophthalmol.*

Symptomatic Subjects	Cone Density	Difference [95%CI]	Cone Density	Difference [95%CI]	Cone Density	Difference [95%CI]	Cone Density	Difference [95%CI]
**Distance/mm**	**0.2**		**0.3**		**0.4**		**0.5**	
**Superior**	30.2	2.13 [0.07 to 3.51]	27.6	1.64 [0.65 to 2.63]	24.0	0.20 [-0.63 to 1.03]	19.3	-2.32 [-3.12 to -1.52]
**Inferior**	31.2	3.25 [1.82 to 4.68]	28.6	2.53 [1.39 to 3.66]	22.6	-1.26 [-2.23 to -0.29]	19.0	-2.02 [-2.82 to -1.22]
**4**								
**Nasal**	26.5	-1.48 [-2.82 to -0.13]	27.9	0.17 [-0.87 to 1.20]	25.3	0.28 [-1.18 to 0.63]	19.1	-4.08 [-4.97 to -3.19]
**Temporal**	24.7	-2.83 [-4.27 to -1.39]	25.9	-1.22 [-2.33 to -0.11]	22.9	-2.41 [-3.33 to -1.49]	20.8	-2.46 [3.24 to -1.69]
**Superior**	26.2	-1.87 [-3.26 to -0.49]	26.7	0.80 [-0.20 to 1.79]	24.7	0.97 [0.14 to 1.80]	18.2	-3.46 [-4.26 to -2.66]
**Inferior**	25.9	-1.97 [-3.40 to -0.54]	24.5	-1.53 [-2.66 to -0.39]	17.0	-6.87 [-7.84 to -5.90]	22.9	1.92 [1.12 to 2.72]
**5**[a]								
**Nasal**	19.6	-8.39 [-9.74 to -7.04]	21.5	-6.27 [-7.31 to -5.23]	21.3	-4.36 [-5.26 to -3.45]	18.0	-5.18 [-6.07 to -4.29]
**Temporal**	15.8	-11.76 [-13.2 to -10.3]	24.1	-3.09 [-4.20 to -1.98]	17.4	-7.97 [-8.89 to -7.05]	17.4	-5.78 [-6.55 to -5.00]
**Superior**	15.6	-12.46 [-13.8 to -11.1]	15.8	10.1 [-11.1 to -9.14]	8.9	-14.9 [-15.7 to -14.1]	13.3	-8.30 [-9.10 to -7.50]
**Inferior**	26.5	-1.41 [-2.84 to 0.01]	29.7	3.70 [2.56 to 4.83]	16.0	-7.86 [-8.83 to -6.89]	10.1	-10.9 [-11.7 to -10.1]
**6**[a]								

© 2025 Liao X et al. *JAMA Ophthalmol.*

Symptomatic Subjects	Cone Density	Difference [95%CI]	Cone Density	Difference [95%CI]	Cone Density	Difference [95%CI]	Cone Density	Difference [95%CI]
**Distance/mm**	**0.2**		**0.3**		**0.4**		**0.5**	
**Nasal**	27.9	-0.07 [-1.42 to 1.28]	33.0	5.24 [4.21 to 6.28]	27.7	2.12 [1.21 to 3.02]	25.1	1.90 [1.01 to 2.80]
**Temporal**	29.8	2.23 [0.79 to 3.67]	27.3	0.18 [-0.93 to 1.29]	28.1	2.78 [1.86 to 3.70]	21.7	-1.55 [-2.32 to -0.78]
**Superior**	28.3	0.30 [-1.08 to 1.68]	27.3	1.40 [0.04 to 2.40]	23.3	0.48 [-1.31 to 0.35]	19.8	-1.79 [-2.59 to -0.99]
**Inferior**	30.2	2.25 [0.82 to 3.68]	23.7	-2.35 [-3.49 to -1.22]	22.1	-1.77 [-2.73 to -0.80]	20.9	0.16 [-0.96 to 0.64]
**7[a]**								
**Nasal**	25.8	-2.18 [-3.52 to -0.83]	26.0	-1.73 [-2.77 to -0.69]	27.2	1.60 [0.70 to 2.51]	23.4	0.20 [-0.69 to 1.10]
**Temporal**	28.0	0.49 [-0.95 to 1.93]	28.0	0.87 [-0.24 to 1.98]	26.4	1.05 [0.13 to 1.97]	25.4	2.18 [1.40 to 2.95]
**Superior**	25.0	-3.00 [-4.38 to -1.62]	25.2	0.72 [-1.71 to 0.28]	23.6	0.15 [-0.98 to 0.68]	20.6	-1.04 [-1.84 to -0.24]
**Inferior**	21.0	-2.87 [-4.30 to 1.45]	27.2	1.16 [0.02 to 2.92]	23.0	-0.83 [-1.80 to 0.14]	21.6	0.59 [-0.22 to 1.39]

a: RLRL therapy < 12 months, cone photoreceptor density calculated in this section only

© 2025 Liao X et al. *JAMA Ophthalmol*.

eFigures

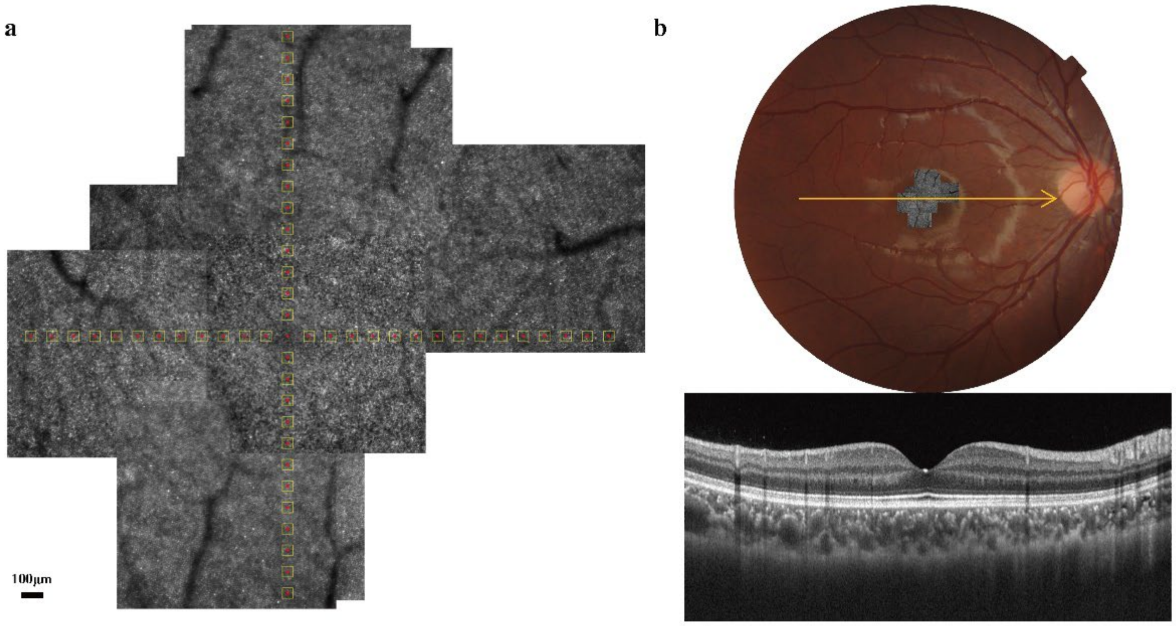

eFigure 1. Montage formation in fundus images and OCT images of a participant. a. Composite montage of AOSLO images depicting the photoreceptor mosaic within a 4° radius of the foveal center. Yellow markers indicate the foveal center and demarcate the cardinal meridians at 100 μm intervals. A representative sampling window (50 μm × 50 μm) is highlighted in yellow. b. Position and actual size of this participant's adaptive optical image in the fundus photo. The yellow arrow represents the direction of the OCT (Fig. c) scan.

OCT: Optical coherence tomography; AOSLO: Adaptive Optics Scanning Laser Ophthalmoscopy.

© 2025 Liao X et al. *JAMA Ophthalmol*.

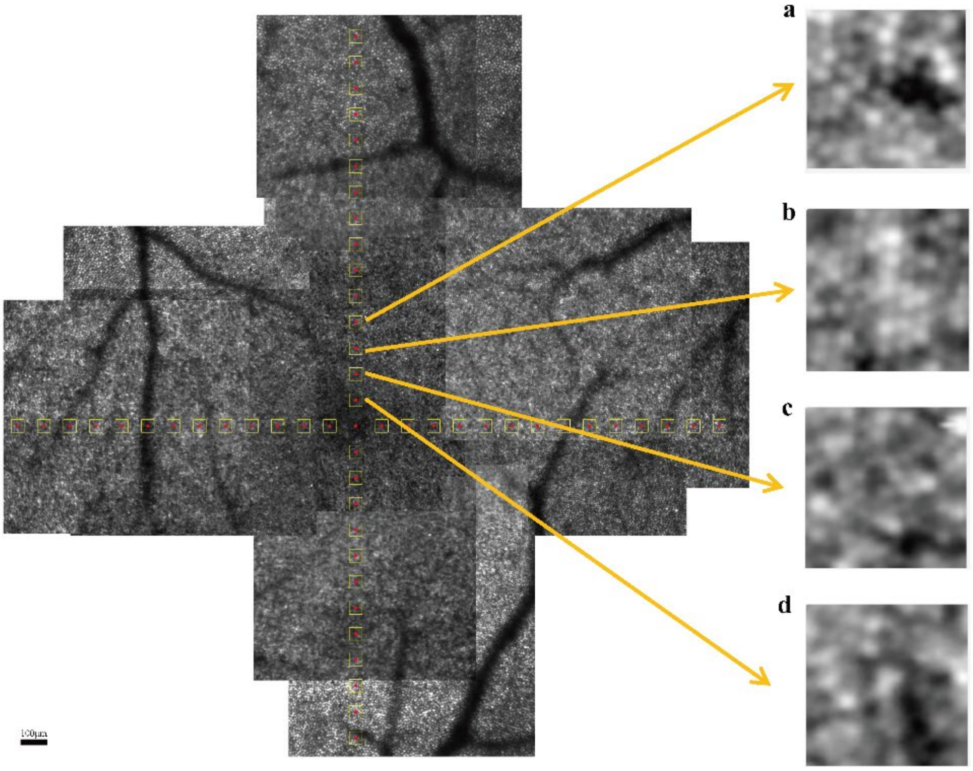

eFigure 2. The right panels a-d show the actual cone images at distances corresponding to different eccentricities on the upper side in a resolution-limited situation. a, 0.4 mm superior to the center fovea; b, 0.30 mm; c, 0.20 mm; d, 0.10 mm.

© 2025 Liao X et al. *JAMA Ophthalmol*.

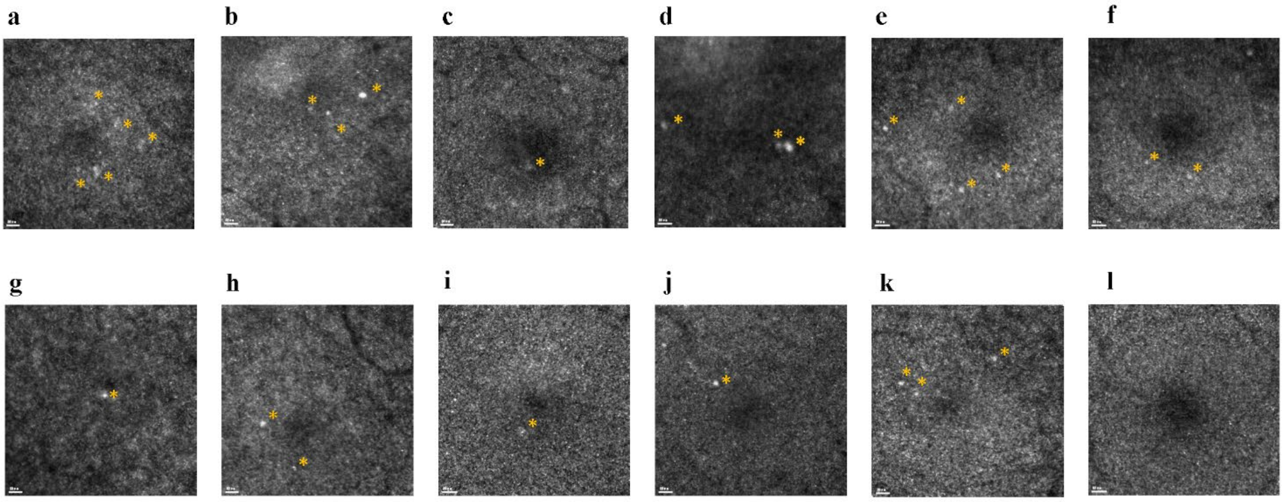

eFigure 3. AOSLO pictures of abnormal low-frequency high-intensity cell clusters in the fovea (Figs. a-k, yellow *) and the normal fovea (Fig.l). Upper panels a-j are from the RLRL group and panels k and l are from the non-RLRL group.

© 2025 Liao X et al. *JAMA Ophthalmol*.

eReferences

1. Ostrin LA, Schill AW. Red light instruments for myopia exceed safety limits. *Ophthalmic and Physiological Optics* 2024; **44**(2): 241-8.
2. Chen Y, Xiong R, Chen X, et al. Efficacy Comparison of Repeated Low-Level Red Light and Low-Dose Atropine for Myopia Control: A Randomized Controlled Trial. *Translational Vision Science & Technology* 2022; **11**(10).
3. Jiang Y, Zhu Z, Tan X, et al. Effect of Repeated Low-Level Red-Light Therapy for Myopia Control in Children. *Ophthalmology* 2022; **129**(5): 509-19.
4. Wang W, Jiang Y, Zhu Z, et al. Axial Shortening in Myopic Children after Repeated Low-Level Red-Light Therapy: Post Hoc Analysis of a Randomized Trial. *Ophthalmol Ther* 2023; **12**(2): 1223-37.
5. Xiong R, Zhu Z, Jiang Y, et al. Sustained and rebound effect of repeated low-level red-light therapy on myopia control: A 2-year post-trial follow-up study. *Clin Exp Ophthalmol* 2022; **50**(9): 1013-24.
6. Zhou W, Liao Y, Wang W, et al. Efficacy of Different Powers of Low-Level Red Light in Children for Myopia Control. *Ophthalmology* 2024; **131**(1): 48-57.
7. Zhou L, Tong L, Li Y, Williams BT, Qiu K. Photobiomodulation therapy retarded axial length growth in children with myopia: evidence from a 12-month randomized controlled trial evidence. *Sci Rep* 2023; **13**(1): 3321.
8. Xiong Y, Liao Y, Zhou W, Sun Y, Zhu M, Wang X. Effectiveness of low-level red light for controlling progression of Myopia in children and adolescents. *Photodiagnosis and Photodynamic Therapy* 2024; **49**: 104267.

© 2025 Liao X et al. *JAMA Ophthalmol*.
