# Supplementary material for: Cone Density Changes After Repeated Low-Level Red Light Treatment in Children With Myopia
Source: JAMA Ophthalmol. 2025 Apr 24;143(6):480–8. doi: 10.1001/jamaophthalmol.2025.0835 (PMC12022868; doi:10.1001/jamaophthalmol.2025.0835)
Supplement: Supplement 2. — Data Sharing Statement. [file jamaophthalmol-e250835-s002.pdf]

Data Sharing Statement

Liao. Cone Density Changes After Repeated Low-Level Red Light Treatment in Children With Myopia. *JAMA Ophthalmol*. Published April 24, 2025. doi:10.1001/jamaophthalmol.2025.0835

Data
Data available: Yes
Data types: Deidentified participant data
How to access data: After the publication of the manuscript, the deidentified participant data will be accessible by contacting us via email at xinyiliao@pku.edu.cn. Reach out to this email address for further details on how to access the data.
When available: With publication

Supporting Documents
Document types: Informed consent form
How to access documents: Following the publication, the informed consent form will be accessible by contacting us via email at xinyiliao@pku.edu.cn. Please reach out to this email address for instructions on how to obtain the informed consent form.
When available: With publication

Additional Information
Who can access the data: Access to the data will only be granted to approved individuals or researchers whose proposed use of the data has been authorized.
Types of analyses: We will disclose this data to researchers studying the relevant content.
Mechanisms of data availability: with investigator support
